# Effects of Pharmacists’ Interventions on Inappropriate Drug Use and Drug-Related Readmissions in People with Dementia—A Secondary Analysis of a Randomized Controlled Trial

**DOI:** 10.3390/pharmacy6010007

**Published:** 2018-01-16

**Authors:** Maria Gustafsson, Maria Sjölander, Bettina Pfister, Jörn Schneede, Hugo Lövheim

**Affiliations:** 1 Department of Pharmacology and Clinical Neuroscience, Umeå University, SE-90187 Umeå, Sweden; maria.sjolander@umu.se (M.S.); bettina.pfister@vll.se (B.P.); jorn.schneede@umu.se (J.S.); 2 Department of Community Medicine and Rehabilitation, Geriatric Medicine, Umeå University, SE-90187 Umeå, Sweden; hugo.lovheim@umu.se

**Keywords:** medication reviews, potentially inappropriate medications, drug-related readmissions, dementia

## Abstract

Age-associated physiological changes and extensive drug treatment including use of potentially inappropriate medications (PIMs) pose a significant risk of drug–drug interactions and adverse drug events among elderly people with dementia. This study aimed at analysing the effects of clinical pharmacists’ interventions on use of PIMs, risk of emergency department visits, and time to institutionalization. Furthermore, a descriptive analysis was conducted of circumstances associated with drug-related readmissions. This is a secondary analysis of data from a randomized controlled intervention study conducted in two hospitals in Northern Sweden. The study included patients (*n* = 460) 65 years or older with dementia or cognitive impairment. The intervention consisted of comprehensive medication reviews conducted by clinical pharmacists as part of a healthcare team. There was a larger decrease in PIMs in the intervention group compared with the control group (*p* = 0.011). No significant difference was found in time to first all-cause emergency department visits (HR = 0.994, 95% CI = 0.755–1.307 *p* = 0.963, simple Cox regression) or time to institutionalization (HR = 0.761, 95% CI = 0.409–1.416 *p* = 0.389, simple Cox regression) within 180 days. Common reasons for drug-related readmissions were negative effects of sedatives, opioids, antidepressants, and anticholinergic agents, resulting in confusion, falling, and sedation. Drug-related readmissions were associated with living at home, heart failure, and diabetes. Pharmacist-provided interventions were able to reduce PIMs among elderly people with dementia and cognitive impairment.

## 1. Introduction

Elderly people are known to be at increased risk of adverse drug events. A higher prevalence of diseases and chronic medical conditions in the elderly population results in extensive drug treatment, which increases the number of possible drug interactions [1]. Furthermore, age-associated physiological changes that alter drug pharmacokinetics and pharmacodynamics lead to an increased susceptibility to adverse drug effects among elderly people [2]. The elderly are often excluded from clinical studies, and drug treatment and dosing is often guided by data from studies of younger people without comorbidities [3].

Elderly people with dementia are even more vulnerable to drug-related problems due, for example, to more pronounced changes in endogenous neurotransmitter concentrations such as acetylcholine and dopamine in the central nervous system (CNS) [4]. Also, prescription rates are high among elderly people with dementia [5], and the choice of drugs is often inappropriate [6]. Studies have reported frequent use of potentially inappropriate medications (PIMs) in this patient group, which raised increasing concerns about the consequences of inappropriate drug use during recent years [6]. Use of PIMs has been associated with increased risk of adverse drug events and hospitalization [7,8,9]. Drug-related causes of hospital admissions are common among elderly people, and especially among elderly people with dementia [10].

We have previously reported the effects of pharmacist interventions on drug-related readmissions in a group of patients 65 years or older with dementia or cognitive impairment [11]. The main finding from this prospective, randomized, controlled trial (RCT) was that the addition of clinical pharmacists to the healthcare team did not reduce the risk of drug-related readmissions during a 180-days follow-up period. However, post hoc and subgroup analyses indicated significant effects of the intervention during the first 30 days after intervention and among people without heart failure.

In the present study, we analysed pre-specified secondary outcome parameters from the RCT: the use of PIMs, risk of emergency department visits, and time to institutionalization. A second aim was to describe more specifically the reasons for drug-related readmissions among this patient group. Finally, possible associations between various demographic and clinical factors and risk of drug-related hospitalizations were analysed.

## 2. Materials and Methods

### 2.1. Subjects and Settings

The prospective RCT comparing standard care with additional pharmacist intervention has been described in detail previously [11]. Briefly, 460 patients aged 65 years or older with dementia or cognitive impairment were included from the acute internal medicine ward and the orthopedic ward at Umeå University Hospital, and from the medicine wards at the county Hospital in Skellefteå, Sweden. Between 9 January 2012, and 2 December 2014, 473 patients were invited to participate in the trial. Thirteen patients declined participation. Patients from all wards were randomly assigned to the intervention or control groups. Persons who deceased before discharge (31 persons) were excluded. The final sample was 429 persons. The main components of the enhanced service provided by the clinical pharmacists to the intervention group included medication reconciliations and comprehensive medication reviews where drug-related problems were identified and orally communicated and discussed in the ward team. The identified drug-related problems have been reported in greater detail by Pfister et al. [12]. The primary outcome, risk of drug-related readmissions, was assessed by an independent and blinded external expert group consisting of one specialist in geriatrics, one specialist in internal medicine, and one clinical pharmacist working in another county. For each participant, the expert group received the drug list, laboratory list, doctors’ notes, and the epicrises from the first admissions and from any readmission(s). The expert group decided whether or not readmissions were drug-related. Cases on which the expert group was unable to reach agreement were discussed by the whole group in order to reach a final decision. The procedure is more described in Gustafsson et al. [11].

### 2.2. Definitions

Six drug-specific quality indicators as defined by the Swedish National Board of Health and Welfare [1] were used to define use of PIMs in this study. Four out of the six selected indicators belong to a group where drug-use should be as low as possible regardless of indication: *anticholinergic drugs* (as defined by the Swedish National Board of Health and Welfare [1]), *propiomazine*, *tramadol*, and *long-acting benzodiazepines*. The two remaining indicators are classified as preparations for which correct and current indication is of particular importance: *antipsychotic drugs* (N05A except lithium) and *Nonsteroidal Anti-Inflammatory Drugs (NSAIDs).* However, since the latter drug groups exhibit high risk of side effects in the present study population, in the present analysis they were treated in the same way as the former indicators, i.e., the number of people using these drugs should be as low as possible, regardless of indication. In the present study, a PIM was defined as exposure to at least one of the drugs mentioned among the six quality indicators. This definition differs from the one used in Gustafsson et al., where inappropriate drugs were added to the group ineffective drugs. Also, different drug-specific indicators defined by the Swedish National Board of Health and Welfare were used in the present study, compared to Gustafsson et al. [11,12].

### 2.3. Procedures

To investigate the effects of pharmacist intervention on the use of PIMs, the number of people with PIMs was measured at index admission (at randomization) and at index discharge in the intervention and control groups. Although randomization and first day of index admission could differ by a few days, the term index admission is used for simplicity. Time to first all-cause emergency department visits (including visits leading to hospitalizations) within 30 and 180 days from index discharge was measured. Time to institutionalization was measured from index discharge, and the follow-up time was 180 days. Additionally, the reasons for all drug-related readmissions as judged by the expert group were described for both groups during 30 days and 180 days after the index discharge. Finally, for analysis of associations between drug-related readmissions and different demographic and clinical factors, the intervention and control group were combined in one sample.

### 2.4. Data Analysis

McNemar’s test without Yates correction was used to compare the number of people with PIMs at admission and discharge. The difference in change in the number of people with PIMs at admission and discharge between intervention and control group was tested by means of a chi-square test. A Cox regression model was used for analysis of the outcome parameter time to first emergency department visit and time to institutionalization in intervention and control group after index discharge. For separate analysis of the specific causes of drug-related readmission, the difference in proportion of the total number of drug-related readmissions in intervention and control group for each cause was tested by means of a chi-square test.

Simple logistic regression analyses were conducted to investigate the association between drug-related readmission and various factors retrieved from the medical record. These factors were gender, age, number of medications at discharge, PIMs at discharge, type of ward, type of living, MMSE, creatinine clearance, and the patients’ medical history. Multiple logistic regression analysis was conducted including age, gender, and significant variables from the simple models. Results are presented as odds ratios (ORs) with 95% confidence intervals (CIs). *p*-values < 0.05 were considered statistically significant. All statistical analyses were carried out using Statistical Package for the Social Sciences (SPSS) for Windows Version 22.0.

### 2.5. Ethical Approval and Trial Registration

Permission for the present study was sought and approved for research without consent in accordance with the Swedish Ethical Review Law (Regional Ethical Review Board in Umeå, Sweden, registration number 2011-148-31M). Trial Registration: clinicaltrials.gov NCT01504672.

## 3. Results

Baseline characteristics between the intervention and control groups were comparable, except that significantly more patients in the intervention group had a history of heart failure (34% vs. 25%, *p* = 0.039) [11].

The number of patients exposed to PIMs is presented in Table 1. In the intervention group, PIMs decreased significantly from 20.3% to 14.2% (*p* = 0.002). Specifically, the use of anticholinergic drugs decreased significantly from 7.1% to 3.3 % (*p* = 0.005) and NSAIDs decreased from 3.3% to 0.9% (*p* = 0.025) between index admission and discharge. However, there was no statistically significant difference in use of long-acting benzodiazepines, propiomazine, tramadol, or antipsychotics between admission and discharge in the intervention group. In contrast, in the control group, there was no statistically significant difference between admission and discharge for any of the selected PIMs when analysed separately. Nevertheless, the overall percentage of PIM use decreased significantly from 20.7% to 18.4% (*p* = 0.025). The decrease in overall number of PIMs in the intervention group was larger than in the control group (*p* = 0.011).

Within 180 days after discharge, 190 all-cause emergency department visits occurred in the intervention group and 184 in the control group. Most of these visits resulted in consecutive hospitalizations (138 in the intervention group and 141 in the control group). During this follow-up period, there was no statistically significant difference in time to first all-cause emergency department visits between the groups (HR = 0.994, 95% CI = 0.755–1.307 *p* = 0.963, simple Cox regression). Likewise, there was no statistically significant difference in time to first all-cause emergency department visits between the groups during the first 30 days after discharge (HR = 0.780, 95% CI = 0.513–1.186 *p* = 0.246, simple Cox regression). During the 180 days of follow-up, 11.6% (17/146) of patients in the intervention group and 15.2% (24/158) of the control group had moved from home to a nursing home (HR = 0.761, 95% CI = 0.409–1.416 *p* = 0.389, simple Cox regression). Adjustment for heart failure (which was more common in the intervention group) [11] did not substantially alter the results for time to all-cause emergency department visits or time to institutionalization (data not shown).

During the first 30 days after discharge, 39 of 80 readmissions (49%) were judged to be drug-related by the expert group, 26 of 46 (57%) readmissions in the control group, and 13 of 34 (38%) in the intervention group (*p* = 0.028). Specific causes of drug-related readmissions are listed in Table 2. The most common drug-related causes of readmissions within 30 days were use of sedatives, opioids, antidepressants, and anticholinergic agents, resulting in sedation, confusion, and falls. Readmissions due to hypoglycemia/hyperglycemia were more common in the intervention group compared to the control group (2 readmissions vs. 0 readmissions, *p* = 0.040). During 180 days of follow-up, 126 of 279 readmissions (45%) were considered drug-related, 68 of 141 (48%) readmissions in the control group, and 58 of 138 (42%) in the intervention group (*p* = 0.32) [11]. The most common reasons for drug-related readmission within 180 days were deterioration of heart failure (16 readmissions in the intervention group vs. 6 readmissions in the control group, *p* = 0.006), followed by overprescribing of antihypertensive and diuretic agents resulting in bradycardia, hypotension, and dehydration. Readmissions because of confusion, sedation, or fall judged to be caused by sedatives, opioids, antidepressants, or anticholinergic drugs were more common in the control group compared to the intervention group (14 readmissions vs. 4 readmissions, *p* = 0.029).

In the total study population, 90 people were readmitted for drug-related reasons within 180 days (Table 3). Simple logistic regression analysis indicated that drug-related readmissions were more common among people consuming a larger number of drugs (OR, 1.09 [95% CI, 1.02–1.16]) and among people living at home (OR, 2.01 [95% CI, 1.13–3.57]). Also, drug-related readmissions were more common among people with heart failure (OR, 2.66 [95% CI, 1.64–4.30]), cardiac arrhythmia (OR, 2.15 [95% CI, 1.32–3.51]), diabetes mellitus (OR, 2.32 [95% CI, 1.41–3.81]), and chronic obstructive pulmonary disease (OR, 2.22 [95% CI, 1.05–4.67]). In the simple logistic regression, there were no statistically significant differences between patients with and without drug-related readmissions with respect to all other factors (gender, age, MMSE, use of PIMs at discharge, or ward type). In a multiple logistic regression model with drug-related readmission as the dependent variable and significant variables from the simple model as independent variables, living at home (OR, 2.51 [95% CI, 1.34–4.70]), heart failure (OR, 2.11 [95% CI, 1.17–3.79]), and diabetes mellitus (OR, 2.03 [95% CI, 1.17–3.50]) remained significant.

## 4. Discussion

Clinical pharmacists’ interventions resulted in a larger reduction of PIMs compared with standard hospital care. No significant differences in the time to first all-cause emergency department visits or time to institutionalization were observed. Furthermore, we noticed that use of sedatives, opioids, antidepressants, and anticholinergic agents resulting in confusion, falling, and sedation, as well as deterioration of heart failure, were common reasons for drug-related readmissions. In the total study population, drug-related readmissions were more common in people living at home, or having heart failure or diabetes mellitus.

The results of this study are in line with previous research demonstrating reduced numbers of PIMs when involving clinical pharmacist services [13,14]. Avoidance of PIMs is just one of many important aspects on which a clinical pharmacist focuses during medication reviews [12]. Nevertheless, it is important to identify these drugs since they have consistently been associated with adverse drug reactions and hospitalization among elderly people [10,15].

In the present study, PIMs such as anticholinergic drugs were judged in some cases as a cause of adverse drug events leading to readmission to hospital, even though no associations with drug-related readmissions were found in the multiple regression models. Most likely, the number of observations was too limited to demonstrate statistically significant differences. Nevertheless, anticholinergic drugs were one of the groups in which there was a decrease following pharmacist intervention. These drugs increase the risk of peripheral symptoms, such as constipation and urinary retention, but also of central nervous system side effects, including confusion and memory impairment [16]. This is of special clinical relevance in elderly people with dementia, as these adverse effects may be more pronounced due to the cholinergic deficits [17]. Furthermore, anticholinergic drugs may also counteract the potential benefits of cholinesterase inhibitors [18]. NSAIDs were another group of drugs that declined significantly in the intervention group. This is a very important drug class to pay attention to since elderly people are at high risk of developing side effects from NSAIDs, such as gastrointestinal bleeding and acute renal failure. NSAIDs may also increase the risk of hypertension and heart failure [19,20,21]. The use of antipsychotic drugs did not decrease in either of the groups. Antipsychotics are considered potentially inappropriate medications as they may increase the risk of falls and are associated with higher mortality [1]. However, since dementia is associated with behaviour and psychological symptoms such as aggression and hallucinations, it is difficult to totally refrain from using antipsychotics in this group of patients. Besides, a few people had chronic psychotic illnesses, which required treatment with antipsychotics. However, close monitoring of effects and side effects is of great importance [1].

In line with the primary outcome from the RCT [11], no significant difference was found in the number of all-cause emergency department visits during 180 days of follow-up. The external expert group did not judge the drug-relatedness of these visits, and thus the contribution of drug-related reasons is not known. During 180 days of follow-up, the most common drug-related reason for hospital readmission was impaired heart failure. It was significantly more common to be readmitted to hospital because of impaired heart failure in the intervention group compared to the control group in the present study. As previously mentioned, significantly more people in the intervention group suffered from heart failure compared to the control group, and additionally, no effect of the intervention could be seen among people with heart failure [11]. Heart failure is a severe clinical condition with high risk of exacerbations. Heart failure is commonly treated with medications such as ACE-inhibitors, aldosterone-antagonists, beta-blockers, and diuretics, however, these drugs also increase the risk of orthostatic hypotension, dizziness, fall, fracture, and also dehydration and hyponatremia. This might have contributed to an increased number of hospitalizations in the present study, and is in line with previous research [22]. Balancing benefits and potential adverse effects of heart failure medications is a difficult task especially in the present study population where adherence problems are common. Even minor cognitive impairments have been found to impact adherence negatively [23], and adherence is crucial to avoid hospitalization among people with heart failure [24,25]. Issues such as the need to restrict liquid intake or self-adjust diuretic dosages can arise. Adherence problems may not only affect heart failure patients, some of the observed readmissions were also due to non-adherence regarding insulin, resulting in hypo- or hyperglycemia. Appropriate dosage and administration of insulin is crucial, and optimal insulin treatment might be especially difficult among people with concomitant cognitive impairment. In the multiple regression models, particularly heart failure, diabetes, and living at home were associated with drug-related readmissions. Consequently, it is especially important to identify people with dementia who are still living at home and are suffering from these diseases. These people deserve special attention in outpatient care to reduce the risk of early readmission. To meet these needs, more complex interventions conducted in collaboration between different healthcare providers in secondary and primary care may be indispensable. Probably an even more complex intervention is needed to impact on time to institutionalization.

Readmissions due to use of central nervous system depressants such as sedatives, opioids, antidepressants, or anticholinergic drugs were more common in the control group compared to the intervention group. A central issue for the clinical pharmacists was to investigate whether the indication was current or suggesting dose-adjustments of these drugs due to for example impaired renal function. Confusion, sedation, and fall were frequent adverse causes of acute readmissions. Our findings are in line with previous research reporting these adverse drug events to be frequently caused by psychotropic drugs [22]. In the present study, in some instances too-high doses of these drugs were prescribed without taking into account impaired renal function. Similar observations on inappropriately high drug doses relative to renal function were made in connection with the patients’ first admissions to hospital [26]. Still, in other cases, adverse drug events were observed despite use of appropriate drugs at adequate doses. In conclusion, these results underscore the necessity to carefully consider any prescription and to closely monitor dosage and effects in this group of elderly people with dementia.

Lack of drug treatment for atrial fibrillation, embolism, and myocardial infarction were other reasons for drug-related readmissions in both the intervention and the control groups. Among elderly people with dementia, it is even more important to weigh risks against benefits of a treatment, and sometimes, physicians decided not to prescribe anticoagulants despite a clinical indication because of risk of for example adherence problems. Other causes of readmissions were persisting infections due to lack of efficacy of antibacterial treatment due to, e.g., interaction problems between concomitant exposure to calcium and ciprofloxacin per os, or because of too low doses of antibiotics.

One limitation of the study is that the primary outcome parameter, drug-related readmissions, is not an objective measure. However, judgments on whether readmissions were due to drug-related problems were made independently and blinded by three experienced clinical experts with different and complimentary professional backgrounds. Moreover, the external expert panel had no involvement whatsoever in the study. As a result, we consider that the experts’ judgments had high quality and validity.

The fact that the clinical pharmacists had been working on the wards before the study was performed, and that patients from the same wards were randomized to both the intervention and the control groups, may have caused a risk of contamination bias. It is possible that the intervention would have had a higher impact if this had not been the case.

Finally, in the present study, PIMs are reported regardless of dose. A person prescribed a PIM at admission but with reduced dosage at discharge still counts as using a PIM.

## 5. Conclusions

Pharmacist-provided intervention significantly reduced the use of potentially inappropriate medications among elderly people with dementia and cognitive impairment. No statistically significant differences in time to first all-cause emergency department visits was observed between the groups within 30 days or 180 days after discharge. Similarly, no statistically significant differences in time to institutionalization were seen between the groups. People living at home and people with heart failure or diabetes mellitus should receive special attention and improved cooperative activity among health care providers to avoid drug-related readmissions.

## Figures and Tables

**Table 1:** Number and percentage of people using potentially inappropriate medications (PIMs) at index admission and at discharge from hospital.

	Intervention Group (*n* = 212)		Control Group (*n* = 217)			
	At Admission	At Discharge	*p*-Value ^b^	At Admission	At Discharge	*p*-Value ^b^
Anticholinergic drugs, *n* (%)	15 (7.1)	7 (3.3)	0.005	12 (5.5)	9 (4.1)	0.083
Propiomazine, *n* (%)	6 (2.8)	5 (2.4)	0.317	6 (2.8)	6 (2.8)	-
Tramadol, *n* (%)	4 (1.9)	3 (1.4)	0.317	5 (2.3)	4 (1.8)	0.317
Long-acting benzodiazepines, *n* (%)	1 (0.5)	1 (0.5)	-	6 (2.8)	6 (2.8)	-
Antipsychotic drug use, *n* (%)	16 (7.5)	16 (7.5)	-	12 (5.5)	13 (6.0)	0.317
NSAID, *n* (%)	7 (3.3)	2 (0.9)	0.025	11 (5.1)	9 (4.1)	0.157
Potentially inappropriate medication ^a^, *n* (%)	43 (20.3)	30 (14.2)	0.002	45 (20.7)	40 (18.4)	0.025

^a^ Defined as exposure to at least one of the following quality indicators: use of anticholinergic drugs, use of propiomazine, use of tramadol, use of long-acting benzodiazepines, use of antipsychotics, or use of NSAID. ^b^ McNemar’s test without Yates correction was used to analyse the data.

**Table 2:** Drug-related cause of readmission within 30 and 180 days after index discharge, as judged by a blinded, external expert group.

	Number of Drug-Related Readmissions within 30 Days			Number of Drug-Related Readmissions within 180 Days		
Specific Cause of Drug-Related Readmission	Intervention Group (*n* = 13)	Control Group (*n* = 26)	*p*-Value	Intervention Group (*n* = 58)	Control Group (*n* = 68)	*p*-Value
Acute renal failure (ADR)					1	0.354
Bleeding due to anticoagulants				3	3	0.842
Confusion, sedation and/or fall due to sedatives, opioids, antidepressants, or anticholinergic drugs		6	0.060	4	14	0.029
COPD exacerbation (non-adherence *)		2	0.305		2	0.188
Dehydration due to diuretics	1		0.152	2	1	0.468
Delusions/hallucinations/paranoia					1	0.354
Diarrhoea due to antibiotic treatment				1		0.277
Digoxin intoxication		1	0.474		1	0.354
Dyspnea (ADR)		1	0.474		1	0.354
Gout due to thiazides		1	0.474		1	0.354
Hyponatremia due to diuretics and selective serotonin reuptake inhibitor therapy				3	2	0.523
Infection due to lack of efficacy (i.e., interaction, wrong drug, wrong dose) or ADR		2	0.305	1	5	0.139
Lack of drug treatment for atrial fibrillation, embolism, myocardial infarction	1	1	0.608	4	2	0.299
Orthostatic hypotension, dizziness, fall, fracture due to antihypertensive drugs	3	3	0.346	9	10	0.899
Pulmonary embolism (ADR)				2		0.123
Reduced general condition (ADR)					1	0.354
Seizure (ADR)	1		0.152	1		0.277
Subileus	1	1	0.608	1	2	0.655
Suboptimal use of drugs (including suboptimal prescribing, nonadherence, interactions) leading to:						
Anemia/hematuri		2	0.305		3	0.105
Angina	2	2	0.455	4	7	0.501
Constipation				1	3	0.391
Deterioration of heart failure	2	3	0.735	16	6	0.006
Hypoglycemia/hyperglycemia	2		0.040	3	1	0.237
Osteoporosis				1		0.277
Pain				1		0.277
Seizure		1	0.474	1		0.277
Stroke/TIA					1	0.354

* None of the inhalers was adapted for people with dementia.

**Table 3:** Characteristics of study population with and without drug-related readmission within 180 days.

	Drug-Related Readmission	Non Drug-Related Readmission	Simple OR (95% CI)	Multiple OR (95% CI)
Cases *n* (%)	90 (21.0)	339 (79.0)		
Women *n* (%)	52 (57.8)	219 (64.6)	0.750 (0.467–1.204)	0.920 (0.540–1.568)
Age mean ± SD	82.3 ± 6.6	83.4 ± 6.6	0.976 (0.942–1.011)	0.978 (0.939–1.018)
Number of medications at discharge ± SD	9.2 ± 3.6	8.2 ± 3.5	1.086 (1.017–1.160)	1.041 (0.967–1.121)
PIM at discharge *n* (%)	14 (15.6)	56 (16.5)	0.931 (0.492–1.762)	
Type of ward				
Medical ward *n* (%)	82 (91.1)	286 (84.4)	ref	
Orthopedic ward *n* (%)	8 (8.9)	53 (15.6)	0.526 (0.241–1.152)	
Type of living				
Nursing home *n* (%)	17 (18.9)	108 (31.9)	ref	
Living at home *n* (%)	73 (81.1)	231 (68.1)	2.008 (1.130–3.568)	2.511 (1.340–4.704)
MMSE (0–30) mean ± SD	20.6 ± 4.3	19.6 ± 4.6	1.049 (0.959–1.148)	
Creatinine clearance (mL/min)	54.3 ± 25.5	55.4 ± 21.7	0.998 (0.987–1.008)	
Medical history				
Heart failure *n* (%)	42 (46.7)	84 (24.8)	2.656 (1.640–4.301)	2.106 (1.172–3.787)
Cardiac arrhythmia *n* (%)	37 (41.1)	83 (24.5)	2.153 (1.323–3.506)	1.500 (0.851–2.645)
Diabetes mellitus *n* (%)	35 (38.9)	73 (21.5)	2.319 (1.411–3.810)	2.026 (1.173–3.501)
Chronic obstructive pulmonary disease *n* (%)	12 (13.3)	22 (6.5)	2.217 (1.052–4.673)	1.656 (0.698–3.928)
Stroke, past *n* (%)	18 (20.0)	78 (23.0)	0.837 (0.471–1.487)	

CI = Confidence interval; MMSE = Mini Mental State Examination (*n* = 157); OR = Odds ratio; PIM = Potentially inappropriate drugs (defined as being prescribed at least one of the following drugs (quality indicators): use of anticholinergic drugs, use of propiomazine, use of tramadol, use of long-acting benzodiazepines, use of antipsychotics, or use of non-steroidal anti-inflammatory drugs (NSAID)); SD = standard deviation. Creatinine clearance was based on P-creatinine applying the Cockcroft–Gault equation. The multiple logistic regression model includes age, gender, and significant variables as independent variables.

## References

[1] The National Board of Health and Welfare (2010). Indikatorer för God Läkemedelsterapi hos Äldre (Indicators for Evaluating the Quality of Older People’s Drug Therapy). http://www.socialstyrelsen.se/publikationer2010/2010-6-29.

[2] Mangoni A.A., Jackson S.H.D. (2004). Age-related changes in pharmacokinetics and pharmacodynamics: Basic principles and practical applications. Br. J. Clin. Pharmacol..

[3] Herrera A.P., Snipes S.A., King D.W., Torres-Vigil I., Goldberg D.S., Weinberg A.D. (2010). Disparate inclusion of older adults in clinical trials: Priorities and opportunities for policy and practice change. Am. J. Public Health.

[4] Moore A., O’Keeffe S. (1999). Drug-Induced Cognitive Impairment in the Elderly. Drugs Aging.

[5] Lau T.D., Mercaldo D.N., Harris T.A., Trittschuh T.E., Shega T.J., Weintraub T.S. (2010). Polypharmacy and Potentially Inappropriate Medication Use Among Community-dwelling Elders With Dementia. Alzheimer Dis. Assoc. Disord..

[6] Sönnerstam E., Sjölander M., Gustafsson M. (2017). An evaluation of the prevalence of potentially inappropriate medications in older people with cognitive impairment living in Northern Sweden using the EU(7)-PIM list. Eur. J. Clin. Pharmacol..

[7] Price S.D., Holman C.D., Sanfilippo F.M., Emery J.D. (2014). Association between potentially inappropriate medications from the Beers criteria and the risk of unplanned hospitalization in elderly patients. Ann. Pharmacother..

[8] Hamilton H., Gallagher P., Ryan C., Byrne S., O’Mahony D. (2011). Potentially inappropriate medications defined by STOPP criteria and the risk of adverse drug events in older hospitalized patients. Arch. Intern. Med..

[9] Skoldunger A., Fastbom J., Wimo A., Fratiglioni L., Johnell K. (2015). Impact of Inappropriate Drug Use on Hospitalizations, Mortality, and Costs in Older Persons and Persons with Dementia: Findings from the SNAC Study. Drugs Aging.

[10] Gustafsson M., Sjölander M., Pfister B., Jonsson J., Schneede J., Lövheim H. (2016). Drug-related hospital admissions among old people with dementia. Eur. J. Clin. Pharmacol..

[11] Gustafsson M., Sjölander M., Pfister B., Jonsson J., Schneede J., Lövheim H. (2017). Pharmacist participation in hospital ward teams and hospital readmission rates among people with dementia: A randomized controlled trial. Eur. J. Clin. Pharmacol..

[12] Pfister B., Jonsson J., Gustafsson M. (2017). Drug-related problems and medication reviews among old people with dementia. BMC Pharmacol. Toxicol..

[13] Spinewine A., Swine C., Dhillon S., Lambert P., Nachega J.B., Wilmotte L., Tulkens P.M. (2007). Effect of a collaborative approach on the quality of prescribing for geriatric inpatients: A randomized, controlled trial. J. Am. Geriatr. Soc..

[14] Gustafsson M., Sandman P.-O., Karlsson S., Isaksson U., Schneede J., Sjölander M., Lövheim H. (2015). Reduction in the use of potentially inappropriate drugs among old people living in geriatric care units between 2007 and 2013. Eur. J. Clin. Pharmacol..

[15] Gosch M., Wortz M., Nicholas J.A., Doshi H.K., Kammerlander C., Lechleitner M. (2014). Inappropriate prescribing as a predictor for long-term mortality after hip fracture. Gerontology.

[16] Lieberman J.A. (2004). Managing anticholinergic side effects. Prim. Care Companion J. Clin. Psychiatry.

[17] Schliebs R., Arendt T. (2011). The cholinergic system in aging and neuronal degeneration. Behav. Brain Res..

[18] Sink K.M., Thomas J., Xu H., Craig B., Kritchevsky S., Sands L.P. (2008). Dual Use of Bladder Anticholinergics and Cholinesterase Inhibitors: Long-Term Functional and Cognitive Outcomes. J. Am. Geriatr. Soc..

[19] Langman M.J., Weil J., Wainwright P., Lawson D.H., Rawlins M.D., Logan R.F., Murphy M., Vessey M.P., Colin-Jones D.G. (1994). Risks of bleeding peptic ulcer associated with individual non-steroidal anti-inflammatory drugs. Lancet.

[20] Bleumink G., Feenstra J., Sturkenboom M., Stricker B. (2003). Nonsteroidal Anti-Inflammatory Drugs and Heart Failure. Drugs.

[21] Heerdink E.R., Leufkens H.G., Herings R.M.C., Ottervanger J.P., Stricker B.H.C., Bakker A. (1998). NSAIDs Associated with Increased Risk of Congestive Heart Failure in Elderly Patients Taking Diuretics. Arch. Intern. Med..

[22] Salvi F., Marchetti A., D’Angelo F., Boemi M., Lattanzio F., Cherubini A. (2012). Adverse Drug Events as a Cause of Hospitalization in Older Adults. Drug Saf..

[23] Hayes T.L., Larimer N., Adami A., Kaye J.A. (2009). Medication Adherence in Healthy Elders. J. Aging Health.

[24] Chin M.H., Goldman L. (1997). Factors contributing to the hospitalization of patients with congestive heart failure. Am. J. Public Health.

[25] Ghali J.K., Kadakia S., Cooper R., Ferlinz J. (1988). Precipitating factors leading to decompensation of heart failure. Traits among urban blacks. Arch. Intern. Med..

[26] Sönnerstam E., Sjölander M., Gustafsson M. (2016). Inappropriate Prescription and Renal Function among Older Patients with Cognitive Impairment. Drugs Aging.

